# Variation in Siderophore Biosynthetic Gene Distribution and Production across Environmental and Faecal Populations of *Escherichia coli*

**DOI:** 10.1371/journal.pone.0117906

**Published:** 2015-03-10

**Authors:** Laura J. Searle, Guillaume Méric, Ida Porcelli, Samuel K. Sheppard, Sacha Lucchini

**Affiliations:** 1 Gut Health and Food Safety, Institute of Food Research, Norwich, United Kingdom; 2 Institute of Life Science, College of Medicine, Swansea University, Swansea, United Kingdom; 3 Department of Zoology, University of Oxford, Oxford, United Kingdom

## Abstract

Iron is essential for *Escherichia coli* growth and survival in the host and the external environment, but its availability is generally low due to the poor solubility of its ferric form in aqueous environments and the presence of iron-withholding proteins in the host. Most *E*. *coli* can increase access to iron by excreting siderophores such as enterobactin, which have a very strong affinity for Fe^3+^. A smaller proportion of isolates can generate up to 3 additional siderophores linked with pathogenesis; aerobactin, salmochelin, and yersiniabactin. However, non-pathogenic *E*. *coli* are also able to synthesise these virulence-associated siderophores. This raises questions about their role in the ecology of *E*. *coli*, beyond virulence, and whether specific siderophores might be linked with persistence in the external environment. Under the assumption that selection favours phenotypes that confer a fitness advantage, we compared siderophore production and gene distribution in *E*. *coli* isolated either from agricultural plants or the faeces of healthy mammals. This population-level comparison has revealed that under iron limiting growth conditions plant-associated isolates produced lower amounts of siderophores than faecal isolates. Additionally, multiplex PCR showed that environmental isolates were less likely to contain loci associated with aerobactin and yersiniabactin synthesis. Although aerobactin was linked with strong siderophore excretion, a significant difference in production was still observed between plant and faecal isolates when the analysis was restricted to strains only able to synthesise enterobactin. This finding suggests that the regulatory response to iron limitation may be an important trait associated with adaptation to the non-host environment. Our findings are consistent with the hypothesis that the ability to produce multiple siderophores facilitates *E*. *coli* gut colonisation and plays an important role in *E*. *coli* commensalism.

## Introduction

Iron is an essential element implicated in many cellular processes such as DNA replication, energy generation and protection from oxidative stress. When oxygen is present, free ferrous iron (Fe^2+^) is rapidly oxidised to the insoluble ferric iron (Fe^3+^). To cope with the reduced bioavailability of ferric iron, bacteria such as *E*. *coli* have evolved mechanisms to scavenge iron molecules in order to maintain their intracellular iron concentration between 10^-7^ and 10^-5^ M [1]. It has been estimated that in order to survive and multiply in most environments, Gram-negative bacteria require 10^5^ to 10^6^ Fe^3+^ ions per generation [2]. The main mechanism of ferric iron uptake in *E*. *coli* involves the synthesis of up to 4 distinct siderophore molecules with very high affinity for iron. After synthesis, siderophores are secreted into the external environment. Siderophore-iron complexes are then imported through specific transporters and degraded to release the iron in the bacterial cytosol [1]. In *E*. *coli*, siderophores have been described as virulence factors in pathogenic strains and are therefore suggested as targets for antibacterial compounds to limit pathogenic growth [3]. However, their presence in commensal strains highlights the need to reassess the role of siderophores in the general ecology of *E*. *coli*, which also includes the external non-host environment, where *E*. *coli* can persist for several weeks [4]. In particular, vegetables, an increasingly recognised secondary reservoir for *E*. *coli* [5], might represent an iron limited environment where siderophore-mediated iron uptake is an important determinant of bacterial fitness [6–8]. In agreement with this hypothesis, gene expression of the enterobactin and salmochelin siderophores was induced in *Salmonella* Typhimurium in alfalfa root exudates [9].

The diversity in siderophore production displayed by the *E*. *coli* species suggests that the distribution of siderophores among a population is shaped by the environmental requirements, as with other traits [10,11]. Taking advantage of our recently described GMB collection, which groups 96 environmental isolates from agricultural plants, mainly grown in the UK [11], we compared siderophore production and distribution in these plant-associated strains with *E*. *coli* isolated from healthy mammalian hosts. We used this approach to investigate the influence of the environment on siderophore production by *E*. *coli*.

## Materials and Methods

### Bacterial strains

The plant-associated *E*. *coli* strains (GMB collection) used in this study have been described previously [11], and mostly comprise strains isolated between 2008 and 2009 in England from the aerial parts of salad crops such as spinach and rocket (76/96). A minority of strains have been isolated from other crops, salad bags and field soil. The ECOR collection includes 61 isolates obtained from the faeces of healthy mammals and 11 isolates from the urine of women with urinary tract infections [12]. The subset of 61 faecal isolates will be referred to as ECOR-F and comprises 29 human and 32 animal isolates mainly isolated in the USA and Europe in the 1980s. The ECOR collection was kindly provided by Beth Whittam (Michigan State University, USA).

### Siderophore production

To assess siderophore production *in vitro*, bacterial colonies were grown on chrome azurol S (CAS) [13] plates for 48h at 37°C with glucose as the carbon source. Colonies of siderophore-producing bacteria grown on this medium are surrounded by a yellow or orange halo. The ability to produce siderophores was quantified by measuring the halo diameter using the ImageJ software (http://rsbweb.nih.gov/ij/index.html). To assess any siderophore-specific differences in diffusion that may influence halo size on CAS agar plates, a liquid CAS assay was performed. Single colonies were grown in modified M9 media with shaking for 16h at 37°C [14]. OD_600_ measurements were performed before cultures were pelleted and supernatants collected [13]. Supernatant from strains positive for siderophore production cause a colour change from blue to orange when mixed with CAS assay solution. This colour change was measured using OD_630_ and percent siderophore units calculated and standardised to culture OD_600_ [13].

### Multiplex PCR

To maximise the sensitivity of the multiplex PCR in detecting siderophore production/receptor genes, ClustalW multiple sequence alignments were performed using publically available *E*. *coli* sequences from EcoCyc and the National Centre for Biotechnology Information (NCBI). Primers were designed to target conserved regions of 4 biosynthetic and 1 receptor genes for all 4 siderophore systems (Table 1). Primers for the salmochelin PCR targeted export and degradation genes within the salmochelin operon as it only has 1 biosynthesis gene. Product sizes were designed to be of different lengths within one system to ensure that bands could be distinguished on a gel. Template DNA was extracted from overnight cultures using the QIAGEN DNeasy Blood and Tissue extraction kit as per manufacturer’s instructions. 10 ng of DNA was used for each multiplex PCR in a 25 μl reaction volume containing 12.5μl Go-Taq Green Master Mix (Promega) and 0.1 mM of each primer. Amplification for each PCR was as follows: 35 cycles at 95°C for 30s, 55°C for 30s, 72°C for 1 min, and 1 cycle at 72°C for 5 min. Yersiniabactin and aerobactin multiplex PCRs had slight alterations, with the annealing temperature raised to 60°C for the yersiniabactin PCR and elongation step shortened to 40 s for the aerobactin PCR.

**Table 1:** Primers used in this study for multiplex and real-time PCR.

	Gene	Sequence (Forward)	Sequence (Reverse)	Product Size
**Primers for multiplex PCR**				
Enterobactin	*entA*	GTGCGCTGTAATGTGGTTTC	CAGAGGCGAGGAACAAAATC	184
	*entB*	GCGACTACTGCAAACAGCAC	TTCAGCGACATCAAATGCTC	382
	*entC*	GACTCAGGCGATGAAAGAGG	TGCAATCCAAAAACGTTCAA	438
	*entE*	CGTAGCGTCGAGATTTGTCA	CCCATCAGCTCATCTTCCAT	776
	*fepA*	TTTGTCGAGGTTGCCATACA	CACGCTGATTTTGATTGACG	349
Salmochelin	*iroB*	CAACCATCGGTTTGACAGTG	GACGTAACACCGCCGAGTAT	166
	*iroC*	TGCCACACAGGATTTTACCA	CTCACTCTGGGTGCAGCATA	388
	*iroD*	GGTAAGCAGTTGTCCGGTGT	GTTACTGCGGCTCCTATTCG	227
	*iroE*	ATCATAACCTCTGCCCAACG	ACCAACCTCCCTTTCGATCT	300
	*iroN*	CTTCCTCTACCAGCCTGACG	GCTCCGAAGTGATCATCCAT	648
Yersiniabactin	*irp1*	AGAGCGGAAATAACCGAACA	GTAAACAGGCCGTGACGATT	221
	*irp2*	CTGGTGATGGTGATGGAAAA	CCATCGCGATAAATTGTCCT	247
	*irp3*	GTATACCTCGCCGGAACAGA	GCCAGCGTTTGTAAGGAACT	177
	*irp4&5*	GCGCCACAAGGACTGATTAT	GTCTCTCCAGCGACCAGAAC	905
	*fyuA*	GGGAATGTGAAACTGCGTCT	CGGGTGCCAAGTTCATAGTT	791
Aerobactin	*iucA*	ATAAGGGAAATAGCGCAGCA	TTACGGCTGAAGCGGATTAC	212
	*iucB*	CCACGAATAGTGACGACCAA	GTTTTTGATGCAGAGCGTGA	339
	*iucC*	ATTTCGGGAAACGCTTCTTT	GTGGTTCCGCTGTATCACCT	158
	*iucD*	TCTTCCTTCAGTCCGGAGAA	TCCTCATTTTTCCTGGCATC	630
	*iutA*	CCAGCCTCAAACTCCATCAT	ACAGCCGACAACTGGACTCT	157
**Primers for real-time PCR**				
Enterobactin	*entC*	CGAGCGTTTTAGCTCCATTC	CCTCTTTCATCGCCTGAGTC	143
Salmochelin	*iroB*	TATACCGGTCGTGATGCAAA	ATACTCGGCGGTGTTACGTC	150
Yersiniabactin	*irp2*	TAAAACTGAAGCCGGGTCAC	CCGTTGTGTCACCAGAAATG	122
Aerobactin	*iucA*	CTGCCGGTCGGATTTATTTA	ATAAGGGAAATAGCGCAGCA	138
RpoB	*rpoB*	GTGGTGAAACCGCATCTTTT	CGATGTACTCAACCGGGACT	138

### RNA extraction

LB overnight cultures were washed with water and diluted to a final OD_600_ of 0.05 in 250 ml flasks containing 25 ml of modified M9 medium without iron addition [14]. The cultures were incubated at 37°C in a shaking incubator until the exponential phase of growth (OD_600_ = 0.2). Synthesis and degradation of RNA were blocked by adding 1/5 volume of stop-solution (90% ethanol/10% phenol) [15]. The RNA was purified using the Promega SV total RNA purification kit according to the manufacturer’s instructions. Quantification of the RNA was performed by measuring the 260 nm absorbance on a Nanodrop 1000 spectrophotometer.

### Real-time PCR

To determine the level of expression of each siderophore system, 5 μg of total RNA from each sample was reverse-transcribed in 20 μl of buffer containing 0.5 mM dNTPs, 0.2 μg random hexamers and 200 U reverse transcriptase (Fermentas). Target mRNAs were then detected by real-time PCR using SYBR Green JumpStart Taq ReadyMix following the manufacturer’s instructions (Sigma). Quantification was performed using the comparative Ct method relative to *rpoB* as an internal standard. The real-time PCR was performed using gene-specific primer pairs designed *in silico* (http://frodo.wi.mit.edu/) to target conserved regions (Table 1). Amplicons were designed to be 100–150 bp in size and their amplification efficiency determined to avoid potential bias. The amplification cycle was as follows: 40 cycles at 94°C for 30s, 60°C for 30s, 72°C for 40s, and 1 cycle at 72°C for 5 min.

## Results

### Comparison of siderophore production in *E*. *coli* populations of plant-associated and faecal isolates from healthy mammals

**Fig 1:**
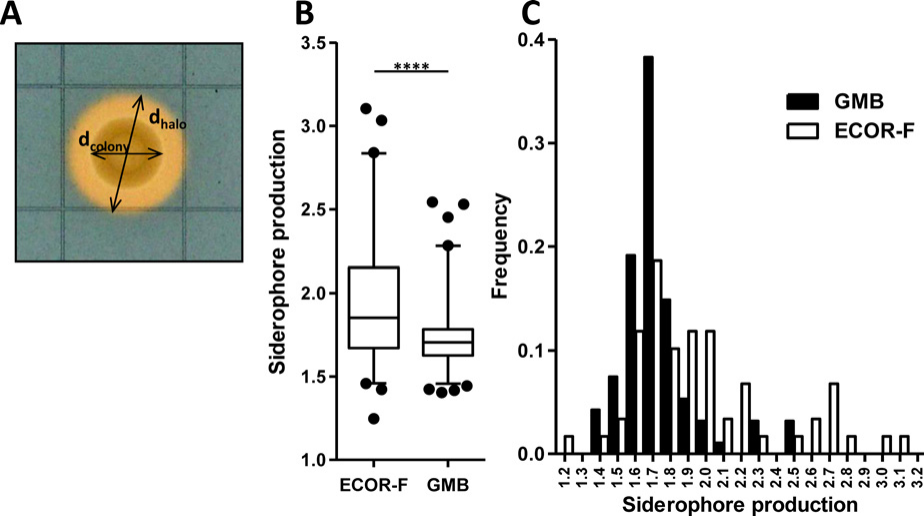
Plant associated *E*. *coli* display lower siderophore production compared to faecal isolates. **A**) Example of siderophore production levels obtained by dividing the halo diameter (d_halo_) by the colony diameter (d_colony_) measured on CAS agar plates. **B**) Box plot showing siderophore production for ECOR-F and GMB. The central rectangle of the plot spans the interquartile range (*IQR*). The segment inside the rectangle shows the median, while the whiskers span the 5–95 percentile. Black circles represent outliers. Statistical significance was determined using the Student *t*-test. ****P<0.0001. **C**) Frequency plot showing siderophore production for both strain collections.

To assess whether environmental adaptation influences the ability of the associated *E*. *coli* populations to generate siderophores, we compared siderophore production of previously described *E*. *coli* strains either isolated from plants (GMB strains) or faecal samples of healthy mammals (ECOR-F subset; S1 Table). The isolates were grown on chrome azurol S (CAS) solid medium and the size of the halo resulting from the colour change following the accumulation of the free indicator dye was measured. Differences in growth rate were normalised by dividing the diameter of the halo by the size of the colony (Fig. 1A). No growth and no halo could be detected for 2 environmental strains (GMB37 and GMB69) and 2 faecal isolates (ECOR29 and ECOR52). Growth was not restored by adding ferrous sulphate to a 100μM final concentration to MM9, suggesting the presence of auxotrophic mutations not related to iron acquisition. These 4 strains were therefore excluded from the siderophore production comparison. Plant-associated strains produced significantly less siderophores (unpaired t-test; t = 4.39, *p*<0.0001) than faecal isolates on the CAS indicator agar medium (Fig. 1B). The frequency distributions of siderophore production for GMB and ECOR-F strains highlighted how a larger (37% vs 11%; Fischer’s exact test, *p*<0.001) proportion of ECOR-F included high siderophore producers (80^th^ percentile) compared to GMB (Fig. 1C).

Previous epidemiological studies have shown that the *iutA*, *iroN* and *fyuA* siderophore receptor genes are more frequently associated with strains belonging to particular phylogenetic groups, with *E*. *coli* isolates belonging to phylogroup B2 generally possessing a greater number of siderophore systems [16–18]. Differences in siderophore production between ECOR-F and GMB could therefore be the simple reflection of the unequal phylogenetic composition of these collections [11]. However, the major *E*. *coli* phylogenetic groups were found to generate similar siderophore levels (One way ANOVA, *p*>0.05; S1 Fig.), indicating that the observed differences are likely to be linked to sample origin rather than phylogenetic composition.

### Design and validation of multiplex PCR for the detection of siderophore utilisation and biosynthetic genes in *E*. *coli*

To determine whether the greater capacity to produce siderophores under iron limitation displayed by the faecal isolates was also reflected at the genome level by a different complement of siderophore biosynthesis genes. We designed a multiplex PCR aimed at detecting both receptor and biosynthesis genes of the four known *E*. *coli* siderophores. We took advantage of the large number of available *E*. *coli* genome sequences to generate multiple alignments and identify suitable conserved regions to design highly sensitive and specific PCR primers (Table 1). The specificity of the multiplex PCR was assessed on the ECOR collection [12], which has been previously monitored by PCR for the presence of siderophore receptor genes [16]. More recently, the gene content of the strains from the ECOR collection has been analysed using multi-genome arrays, which cover the entire enterobactin, aerobactin and salmochelin loci [19].

We found a good correlation with the array data with 96.7% (523/541) of positive and 96.1% (518/539) of negative PCR signals matching both datasets (S2 Table). Results on the presence/absence of the yersiniabactin locus matched previously PCR-based published data in 97.2% (70/72) of cases [20].

Published PCR-based data on the distribution of siderophore receptor genes supported our results when in conflict with the array data (full details are provided in S2 Table). However, our observations that ECOR02 and ECOR67 respectively lack the aerobactin and salmochelin loci were not supported. To exclude the possibility of a sample mix up, we took advantage of existing CRISPR sequence data that can differentiate between the different ECOR strains [21]. The DNA sequences of the CRISPR2.1 and CRISPR2.3 regions of ECOR02 and ECOR67 matched those found in the databases, thus confirming the identity of the strains. We also found differences for 2 strains (ECOR11 and ECOR72) with previously published PCR-based data in the distribution of the yersiniabactin locus (S2 Table) [20] despite the fact that both CRISPR sequences confirmed the identity of these strains. The discrepancies we have identified reflect previous observations that the phenotype and genotype of ECOR strains can differ between laboratories [22], and highlight the importance of characterising the strains used in independent studies.

### Distribution of the aerobactin, enterobactin, salmochelin and yersiniabactin loci in plant and faecal *E*. *coli* isolates

The distribution of the siderophore biosynthetic loci in plant-associated *E*. *coli* was assessed by performing the validated multiplex PCRs on 96 strains from the GMB collection (S3 Table) [11]. Importantly, the siderophore receptor genes were always detected alongside the corresponding biosynthetic genes (133/133, S3 Table). This observation highlights the sensitivity of our approach and indicates that each siderophore locus is generally evolutionarily maintained as a complete unit.

**Fig 2:**
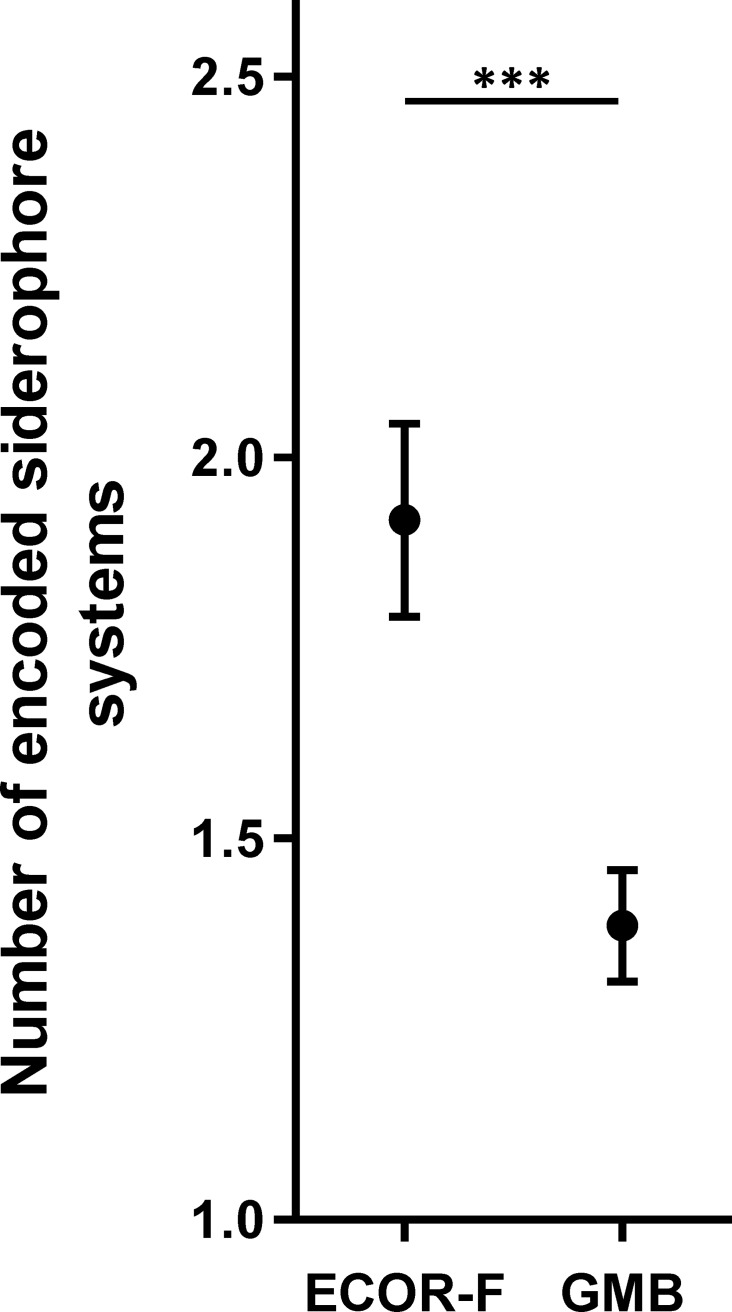
Plant associated *E*. *coli* isolates encode fewer siderophore production systems than faecal isolates at the population level. The graph displays the mean number of detected siderophore systems for the GMB and ECOR-F strain collections. Error bars display the standard error of the mean. Statistical significance was determined using the Student *t*-test. ***P<0.001.

The prevalence of siderophore production genes observed for the strains isolated from plants was compared with that of the healthy host isolates from the ECOR collection (ECOR-F). The results showed that, in accordance with siderophore production data, plant-associated *E*. *coli* generally possessed fewer siderophore production and uptake systems (Fig. 2). Yersiniabactin and aerobactin were found in significantly lower proportions in plant-associated *E*. *coli* (Table 2). This is also reflected by a significant difference in the proportion of strains only possessing the enterobactin locus; 76% (73/96) and 42.6% (26/61) for GMB and ECOR-F respectively (Fischer’s exact test, *p*<0.0001, S2 and S3 Tables).

**Table 2:** Distribution of siderophore biosynthetic systems in the genome of plant and host-associated *E*. *coli* populations.

Siderophore	Proportion of detected systems (%)			Statistical significance^a^		
	GMB (n = 96)	ECOR-F (n = 61)	Faecal^b^ (n = 618–1042)^c^	GMB vs ECOR-F	GMB vs Faecal	ECOR-F vs Faecal
**Aerobactin**	5	25	29	<0.001	<0.001	NS^d^
**Yersiniabactin**	19	48	35	<0.001	<0.01	NS
**Salmochelin**	16	20	21	NS	NS	NS

^a^Significance tests were performed using the Fisher’s exact test. The Benjamini and Hochberg False discovery rate method was used to correct for multiple comparisons.

^b^The data relative to healthy humans were obtained from the literature (see text for references).

^c^The range of isolates tested reflects the fact that not every study included analysed the distribution of all the siderophore systems (aerobactin, n = 1042; yersiniabactin, n = 618; salmochelin, n = 808).

^d^NS: no statistical significance detected.

To expand our comparison beyond ECOR-F, assuming that the presence of the receptor genes generally correlates with the ability to synthesise the corresponding siderophores, we investigated the available recent literature for data describing the distribution of siderophore receptor genes in faecal and rectal *E*. *coli* isolates obtained from healthy humans [17,18,23–30]. Published data was consistent with data obtained for ECOR-F, confirming the narrower distribution of the aerobactin and yersiniabactin loci among the plant-associated isolates (Table 2).

Our results suggest that high siderophore production might be linked to the presence of the aerobactin and/or yersiniabactin loci. Accordingly, the presence of the aerobactin synthesis genes was nearly always associated with strong siderophore production (Table 3). However, no correlation between siderophore production and the presence of the yersiniabactin locus could be observed (Table 3). To exclude any potential effect linked to siderophore-specific differences in diffusion through the agar, the link between aerobactin and strong *in vitro* siderophore production was confirmed by assaying a representative subset of strains (n = 33) in liquid CAS medium (S2A Fig.). The liquid assays also reflected the CAS agar assays in showing lower siderophore production for the plant-associated strains (S2B Fig.).

**Table 3:** Distribution of siderophore biosynthetic systems relative to siderophore production.

	*top 25%*	*25–50%*	*50–75%*	*bottom 25%*
**Aerobactin (%)**	42***	5	3	3
**Yersiniabactin (%)**	42	24	32	31
**Salmochelin (%)**	26*	21	13	5

Significance tests were performed comparing the top and bottom quartiles using the Fischer’s exact test. The Benjamini and Hochberg False discovery rate method was used to correct for multiple comparisons.

*P<0.05,

***P<0.001.

### Strain-specific diversity in siderophore gene expression under low iron conditions

**Fig 3:**
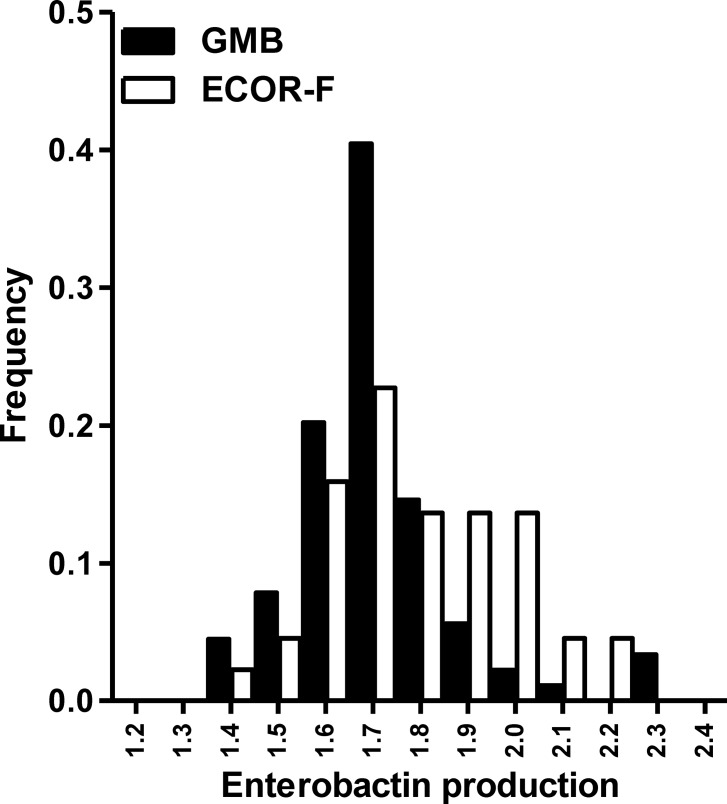
Plant associated *E*. *coli* display lower enterobactin production compared to faecal isolates. Frequency plot comparing siderophore production of GMB and ECOR-F strains only encoding the enterobactin siderophore locus. Sample sizes in the data sets were *n*_GMB_ = 69; *n*_ECOR-F_ = 28.

**Fig 4:**
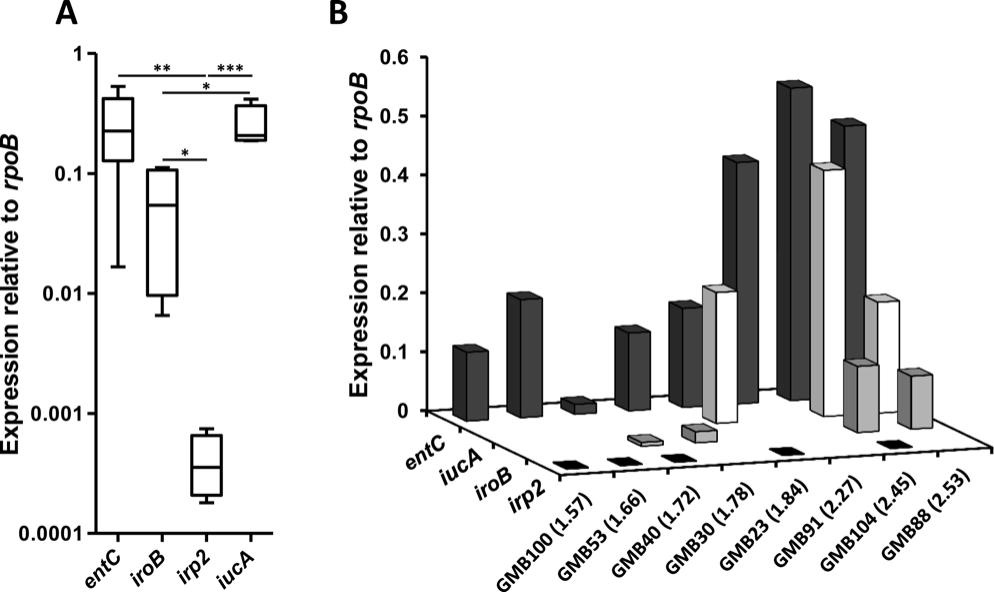
Gene expression level of siderophore biosynthetic genes in plant-associated *E*. *coli*. The expression of one biosynthetic gene was determined for each siderophore locus, when present, in 8 GMB strains; *entC* (enterobactin), *iroB* (salmochelin), *irp2* (yersiniabactin), *iucA* (aerobactin). The strains analysed were GMB23 (*entC*, *irp2*, *iucA*), GMB30 (*entC*, *iroB*), GMB40 (*entC*, *iroB*, *irp2*), GMB53 (*entC*, *irp2*), GMB88 (*entC*, *iroB*, *iucA*), GMB91 (*entC*), GMB100 (*entC*, *irp2*) and GMB104 (*entC*, *iroB*, *irp2*, *iucA*). **A**) Box plot showing the gene expression levels of *entC*, *iroB*, *irp2*, and *iucA* relative to the internal reference *rpoB*. The central rectangle of the plot spans the interquartile range (*IQR*). The segment inside the rectangle shows the median, while the bars above and below show the location of the maximum and minimum, respectively. Statistical significance was determined using the Student *t*-test. In case of multiple tests, the significance of individual *t*-tests was determined using the Benjamini and Hochberg False discovery rate method; *P<0.05, **P<0.01, ***P<0.001. **B**) To visualise the link between gene expression and siderophore production as measured on CAS agar plates, expression levels are shown for each individual strain ranked from low to high producer. Siderophore production is indicated in brackets after the corresponding strain name.

The differential distribution of siderophore loci was not sufficient to fully explain the difference in siderophore production between GMB and ECOR-F isolates. Strains only possessing the enterobactin locus displayed a wide range of siderophore production levels, with significant differences in enterobactin production, observable between plant-associated and faecal isolates at the population level (unpaired t-test; t = 3.13, p = 0.002, Fig. 3). This suggested widespread differences in the regulation of siderophore production between individual *E*. *coli* strains, and thus prompted the investigation of the possible role of differences in gene regulation of enterobactin and the 3 other siderophore systems. We examined the expression levels of one biosynthesis gene for each siderophore in 8 environmental strains displaying a range of siderophore production abilities under low iron conditions. Analysis of the mRNA levels by real-time PCR showed that enterobactin and aerobactin were the most highly expressed systems, both displaying 4 and 600-fold higher expression levels than salmochelin and yersiniabactin, respectively (Fig. 4A). Importantly, enterobactin as well as salmochelin displayed a wide range of different expression levels, which strongly correlated with total siderophore production (Fig. 4B).

In contrast, aerobactin and yersiniabactin displayed a much narrower range of expression, high and very low respectively in all strains tested. This provides a possible explanation for the strong link between presence of the aerobactin locus and high siderophore production in the CAS assays (Table 3, S2A Fig.). Conversely, the generally low yersiniabactin gene expression is in good agreement with the absence of correlation between the yersiniabactin locus and siderophore production levels.

## Discussion

Iron acquisition is vital to microbial physiology and host colonisation. Production of the enterobactin siderophore has been shown to be important for the colonisation of the healthy GI-tract by *E*. *coli* [31]. However, the ability to synthesise additional siderophores is generally associated with virulence, particularly for extraintestinal pathogenic *E*. *coli* that have reached the urinary tract or the blood stream [1,32,33]. The observation that many commensal *E*. *coli* isolates can produce these virulence-associated siderophores suggests another possible role in the ecology of *E*. *coli*, perhaps including persistence outside animal hosts [4]. Reasoning that a particular bacterial function will be more prevalent in the environment where it provides greater fitness advantage, we compared the siderophore production and distribution of siderophore biosynthetic and receptor genes in non-pathogenic *E*. *coli* isolated from plants or mammalian hosts. This population-wide approach has previously been used to identify environment-specific differences in *E*. *coli* carbon source utilisation and extra-cellular matrix production [11].

We found that plant-associated isolates excrete significantly lower amounts of siderophores compared to faecal isolates when grown under iron limiting conditions. Analysis of the distribution of the 4 siderophore biosynthetic systems in both *E*. *coli* populations showed that the aerobactin and yersiniabactin loci were less prevalent in the *E*. *coli* strains isolated from plants. The correlation between genotype and total siderophore production suggested that the greater ability of faecal isolates to generate siderophores may be partly linked to their greater association with the aerobactin locus. Interestingly, the aerobactin biosynthesis genes are often plasmid-encoded [1,34]. This could indicate that the lower frequency of the aerobactin locus among the GMB isolates might be linked to differences in plasmid carriage. When the comparison was restricted to strains only able to synthesise enterobactin, the difference in siderophore production was still significant between plant and faecal isolates, suggesting that diversification in the regulatory response to iron limitation may also be an important environmental adaptive trait. The possible role of differential expression of enterobactin in *E*. *coli* adaptation mirrors previously observed differences in yersiniabactin and aerobactin synthesis between single-patient urinary and rectal *E*. *coli* isolates [35].

Our results suggest that the environment influences the distribution of siderophore production genes among the associated *E*. *coli* populations, and that the gut selects for *E*. *coli* commensal strains with multiple siderophore systems and with the ability to generate higher siderophore levels under iron limitation. In contrast, the plant environment seems to exert a weaker selective pressure on *E*. *coli* to maintain a diverse siderophore production, probably as a consequence of differences in iron source availability compared to the gut. In association with plants, *E*. *coli* could rely on alternative iron acquisition systems such as the direct uptake of ferrichrome or ferric citrate, molecules which can be found both in rhizosphere and phyllosphere [36–38]. It would therefore be interesting to expand this type of analysis to *E*. *coli* populations associated with other plant-hosts and climates to determine whether our observations can be generalised. It is also important to note that the differences in geography and time of isolation between GMB and ECOR-F could be potential confounding factors in our study. However, geography explains only 2% of the genetic diversity between the ECOR strains isolated in Europe and the USA [39]. Similarly, the time of isolation has probably had little impact on the distribution of siderophore receptor genes as more recent studies of human commensal *E*. *coli* have reported comparable gene distribution to ECOR-F (Table 2).

Without excluding a role outside the host, production of aerobactin and yersiniabactin might therefore be important for gut colonisation by commensal *E*. *coli*. This hypothesis is supported by the observation that in contrast to transient *E*. *coli* clones, resident isolates which could be detected in the GI-tract of the same individual over 3 months or more were enriched for aerobactin biosynthesis genes [40]. Similarly, it is possible that yersiniabactin provides a fitness advantage to commensal *E*. *coli* since the rapid spread of the yersiniabactin locus in both virulent and commensal *E*. *coli* implies a role beyond pathogenesis [4,16,20].

Despite the availability of information about siderophore-mediated iron acquisition and the importance of enterobactin for *E*. *coli* gut colonisation [31], it is not clear how having higher and more diverse siderophore production benefits gut commensal *E*. *coli*. It has been shown that the 4 siderophores encoded by *E*. *coli* Nissle 1917 are differently affected by environmental factors such as pH and carbon source [41]. An expanded siderophore repertoire would therefore make a cell more competitive in acquiring iron in the spatially and temporally heterogeneous environment found in the gut. Strains possessing several siderophore systems could also have an advantage in the densely populated gut environment when in competition with bacteria that utilise a narrower range of siderophores.

The metabolic cost of generating siderophores is significant and has a clear impact on the *E*. *coli* metabolome [42]. Indeed, it has been shown that under specific circumstances bacteria can lose the ability to make siderophores while retaining the machinery for acquiring those produced by other strains, thus opening the possibility of cheating [43,44]. However, we observed that the siderophore receptor genes were always detected alongside the corresponding biosynthetic genes. This suggests that the natural environment selects against *E*. *coli* cheaters. One possible explanation might be the poor diffusion of siderophores in viscous media such as mucus [45], where *E*. *coli* is thought to reside, making it less likely for a cheater to be in contact with significant amounts of the required siderophore. This hypothesis is supported by the observation that enterobactin hyperexcretion makes catecholate transport-deficient *E*. *coli* iron-starved *in vivo* despite retaining the ability to import ferric iron through other systems [31]. This phenomenon could be explained by the entrapment of enterobactin around mucus-embedded cells, thus making the iron inaccessible to other acquisition systems.

In summary, our data suggest that the ability to produce yersiniabactin and aerobactin is associated with the colonisation of the gut by *E*. *coli*. The conditions under which each siderophore system is expressed and influences *E*. *coli* fitness in the GI-tract requires further investigation, as does the extent to which our findings can be extended to the persistence of pathogenic *E*. *coli*, such as those strains causing infections of the urinary tract, for which the physiologically normal gut is a likely reservoir [46,47].

## Supporting Information

S1 Fig Phylogenetic distribution of siderophore production. Box plots showing siderophore production by GMB and ECOR-F strains split according to the major *E*. *coli* phylogenetic groups. The central rectangle of the plot spans the interquartile range (*IQR*). The segment inside the rectangle shows the median, while the whiskers span the 5–95 percentile. Black circles represent outliers. Statistical significance was determined using the Student *t*-test. No significant differences were detected.
(PDF)

S2 Fig Siderophore production of GMB and ECOR-F strains as assessed by liquid CAS assay. Box plots showing siderophore production in liquid CAS for a subset of strains (n = 33) to visualise differences in siderophore production of **A**) strains with or without the aerobactin biosynthesis locus, and **B**) GMB and ECOR-F. The central rectangle of the plot spans the interquartile range (*IQR*). The segment inside the rectangle shows the median, while the whiskers span the 5–95 percentile. Black circles represent outliers. Statistical significance was determined using the Student *t*-test; *P<0.05, ****P<0.0001.
(PDF)

S1 Table Siderophore production of GMB and ECOR-F strains as assessed by CAS agar assay. The table displays the ratios between colony and halo diameter as measured on chrome azurol S agar plates for each GMB and ECOR-F strain.
(PDF)

S2 Table Distribution of enterobactin, aerobactin, yersiniabactin and salmochelin loci in the ECOR collection as assessed by multiplex PCR. The table shows presence/absence of the genes encoded in siderophore production loci in *E*. *coli* isolates from the ECOR strain collection.
(PDF)

S3 Table Distribution of enterobactin, aerobactin, yersiniabactin and salmochelin loci in the GMB collection as assessed by multiplex PCR. The table shows presence/absence of the genes encoded in siderophore production loci in *E*. *coli* isolates from the GMB strain collection.
(PDF)

## References

[1] Garenaux A, Caza M, Dozois CM (2011) The Ins and Outs of siderophore mediated iron uptake by extra-intestinal pathogenic *Escherichia coli* . Vet Microbiol 153: 89–98. 10.1016/j.vetmic.2011.05.023 21680117

[2] Braun V, Killmann H (1999) Bacterial solutions to the iron supply problem. Trends Biochem Sci 24: 104–109. 10203757 10.1016/s0968-0004(99)01359-6

[3] Miethke M, Marahiel MA (2007) Siderophore-based iron acquisition and pathogen control. Microbiol Mol Biol Rev 71: 413–451. 17804665 10.1128/MMBR.00012-07 PMC2168645

[4] van Elsas JD, Semenov AV, Costa R, Trevors JT (2011) Survival of *Escherichia coli* in the environment: fundamental and public health aspects. ISME J 5: 173–183. 10.1038/ismej.2010.80 20574458 PMC3105702

[5] Beuchat LR (2002) Ecological factors influencing survival and growth of human pathogens on raw fruits and vegetables. Microb Infect 4: 413–423. 10.1016/s1286-4579(02)01555-1 11932192

[6] Cornelis P (2010) Iron uptake and metabolism in *pseudomonads* . Appl Microbiol Biotechnol 86: 1637–1645. 10.1007/s00253-010-2550-2 20352420

[7] Diallo S, Crepin A, Barbey C, Orange N, Burini J-F, et al (2011) Mechanisms and recent advances in biological control mediated through the potato rhizosphere. FEMS Microbiol Ecol 75: 351–364. 10.1111/j.1574-6941.2010.01023.x 21204870

[8] Jurkevitch E, Hadar Y, Chen Y (1992) Differential siderophore utilization and iron uptake by soil and rhizospheric bacteria. Appl Environ Microbiol 58: 119–124. 16348618 10.1128/aem.58.1.119-124.1992 PMC195181

[9] Hao L-y, Willis DK, Andrews-Polymenis H, McClelland M, Barak JD (2012) Requirement of siderophore biosynthesis for plant colonization by *Salmonella enterica* . Appl Environ Microbiol 78: 4561–4570. 10.1128/AEM.07867-11 22522683 PMC3370490

[10] Bergholz PW, Noar JD, Buckley DH (2011) Environmental patterns are imposed on the population structure of *Escherichia coli* after fecal deposition. Appl Environ Microbiol 77: 211–219. 10.1128/AEM.01880-10 21075897 PMC3019742

[11] Meric G, Kemsley EK, Falush D, Saggers EJ, Lucchini S (2013) Phylogenetic distribution of traits associated with plant colonization in *Escherichia coli* . Environ Microbiol 15: 487–501. 10.1111/j.1462-2920.2012.02852.x 22934605

[12] Ochman H, Selander RK (1984) Standard reference strains of *Escherichia coli* from natural populations. J Bacteriol 157: 690–693. 6363394 10.1128/jb.157.2.690-693.1984 PMC215307

[13] Payne SM (1984) Detection, isolation, and characterization of siderophores. Methods Enzymol 235: 329–344. 10.1016/0076-6879(94)35151-1 8057905

[14] Watts RE, Totsika M, Challinor VL, Mabbett AN, Ulett GC, et al (2012) Contribution of siderophore systems to growth and urinary tract colonization of asymptomatic bacteriuria *Escherichia coli* . Infect Immun 80: 333–344. 10.1128/IAI.05594-11 21930757 PMC3255690

[15] Tedin K, Blasi U (1996) The RNA chain elongation rate of the lambda late mRNA is unaffected by high levels of ppGpp in the absence of amino acid starvation. J Biol Chem 271: 17675–17686. 8663373 10.1074/jbc.271.30.17675

[16] Johnson JR, Delavari P, Kuskowski M, Stell AL (2001) Phylogenetic distribution of extraintestinal virulence-associated traits in *Escherichia coli* . J Infect Dis 183: 78–88. 11106538 10.1086/317656

[17] Johnson JR, Owens K, Gajewski A, Kuskowski MA (2005) Bacterial characteristics in relation to clinical source of *Escherichia coli* isolates from women with acute cystitis or pyelonephritis and uninfected women. J Clin Microbiol 43: 6064–6072. 16333100 10.1128/JCM.43.12.6064-6072.2005 PMC1317206

[18] White AP, Sibley KA, Sibley CD, Wasmuth JD, Schaefer R, et al (2011) Intergenic sequence comparison of *Escherichia coli* isolates reveals lifestyle adaptations but not host specificity. Appl Environ Microbiol 77: 7620–7632. 10.1128/AEM.05909-11 21908635 PMC3209187

[19] Jackson SA, Patel IR, Barnaba T, LeClerc JE, Cebula TA (2011) Investigating the global genomic diversity of *Escherichia coli* using a multi-genome DNA microarray platform with novel gene prediction strategies. BMC Genomics 12: 349 10.1186/1471-2164-12-349 21733163 PMC3146454

[20] Schubert S, Darlu P, Clermont O, Wieser A, Magistro G, et al (2009) Role of intraspecies recombination in the spread of pathogenicity islands within the *Escherichia coli* species. PLoS Path 5: e1000257. 10.1371/journal.ppat.1000257 PMC2606025 19132082

[21] Diez-Villasenor C, Almendros C, Garcia-Martinez J, Mojica FJM (2010) Diversity of CRISPR loci in *Escherichia coli* . Microbiology-SGM 156: 1351–1361. 10.1099/mic.0.036046-0 28206910

[22] Johnson JR, Delavari P, Stell AL, Prats G, Carlino U, et al (2001) Integrity of archival strain collections: the ECOR collection. ASM News 67: 288–289.

[23] Hilali F, Ruimy R, Saulnier P, Barnabe C, Lebouguenec C, et al (2000) Prevalence of virulence genes and clonality in *Escherichia coli* strains that cause bacteremia in cancer patients. Infect Immun 68: 3983–3989. 10858212 10.1128/iai.68.7.3983-3989.2000 PMC101677

[24] Johnson JR, Johnston B, Kuskowski MA, Nougayrede J-P, Oswald E (2008) Molecular epidemiology and phylogenetic distribution of the *Escherichia coli pks* genomic island. J Clin Microbiol 46: 3906–3911. 10.1128/JCM.00949-08 18945841 PMC2593299

[25] Kudinha T, Kong F, Johnson JR, Andrew SD, Anderson P, et al (2012) Multiplex PCR-based reverse line blot assay for simultaneous detection of 22 virulence genes in uropathogenic *Escherichia coli* . Appl Environ Microbiol 78: 1198–1202. 10.1128/AEM.06921-11 22156422 PMC3272995

[26] Lee S, Yu JK, Park K, Oh E-J, Kim S-Y, et al (2010) Phylogenetic groups and virulence factors in pathogenic and commensal strains of *Escherichia coli* and their association with *bla*(CTX-M). Ann Clin Lab Sci 40: 361–367. 20947811

[27] Mao B-H, Chang Y-F, Scaria J, Chang C-C, Chou L-W, et al (2012) Identification of *Escherichia coli* genes associated with urinary tract infections. J Clin Microbiol 50: 449–456. 10.1128/JCM.00640-11 22075599 PMC3264149

[28] Nowrouzian F, Hesselmar B, Saalman R, Strannegard IL, Aberg N, et al (2003) *Escherichia coli* in infants' intestinal microflora: Colonization rate, strain turnover, and virulence gene carriage. Pediatr Res 54: 8–14. 12700366 10.1203/01.PDR.0000069843.20655.EE

[29] Unno T, Han D, Jang J, Widmer K, Ko G, et al (2011) Genotypic and phenotypic trends in antibiotic resistant pathogenic *Escherichia coli* isolated from humans and farm animals in South Korea. Microbes Environ 26: 198–204. 21558676 10.1264/jsme2.me10194

[30] Vollmerhausen TL, Ramos NL, Guendogdu A, Robinson W, Brauner A, et al (2011) Population structure and uropathogenic virulence-associated genes of faecal *Escherichia coli* from healthy young and elderly adults. J Med Microbiol 60: 574–581. 10.1099/jmm.0.027037-0 21292854

[31] Pi H, Jones SA, Mercer LE, Meador JP, Caughron JE, et al (2012) Role of catecholate siderophores in Gram-negative bacterial colonization of the mouse gut. PLoS One 7: e50020 10.1371/journal.pone.0050020 23209633 PMC3510177

[32] Johnson JR, Clermont O, Menard M, Kuskowski MA, Picard B, et al (2006) Experimental mouse lethality of *Escherichia coli* isolates, in relation to accessory traits, phylogenetic group, and ecological source. J Infect Dis 194: 1141–1150. 16991090 10.1086/507305

[33] Caza M, Lepine F, Milot S, Dozois CM (2008) Specific roles of the *iro*BCDEN genes in virulence of an avian pathogenic *Escherichia coli* O78 strain and in production of salmochelins. Infect Immun 76: 3539–3549. 10.1128/IAI.00455-08 18541653 PMC2493193

[34] Vokes SA, Reeves SA, Torres AG, Payne SM (1999) The aerobactin iron transport system genes in *Shigella flexneri* are present within a pathogenicity island. Mol Microbiol 33: 63–73. 10411724 10.1046/j.1365-2958.1999.01448.x

[35] Henderson JP, Crowley JR, Pinkner JS, Walker JN, Tsukayama P, et al (2009) Quantitative metabolomics reveals an epigenetic blueprint for iron acquisition in uropathogenic *Escherichia coli* . PLoS Path 5: e1000305. 10.1371/journal.ppat.1000305 PMC2637984 19229321

[36] Crowley DE (2007) Microbial siderophores in the plant rhizosphere; Barton LL, Abadia J, editors. 169–198 p.

[37] Reid RK, Reid CPP, Powell PE, Szaniszlo PJ (1984) Comparison of siderophore concentrations in aqueous extracts of rhizosphere and adjacent bulk soils. Pedobiologia 26: 263–266.

[38] Devos CR, Lubberding HJ, Bienfait HF (1986) Rhizosphere acidification as a response to iron-deficiency in bean-plants. Plant Physiol 81: 842–846. 16664912 10.1104/pp.81.3.842 PMC1075437

[39] Miller RD, Hartl DL (1986) Biotyping confirms a nearly clonal population-structure in *Eschericha coli* . Evolution 40: 1–12. 28564121 10.1111/j.1558-5646.1986.tb05712.x

[40] Nowrouzian F, Adlerberth I, Wold PE (2001) P fimbriae, capsule and aerobactin characterize colonic resident *Escherichia coli* . Epidemiol Infect 126: 11–18. 11293669 10.1017/s0950268801005118 PMC2869660

[41] Valdebenito M, Crumbliss AL, Winkelmann G, Hantke K (2006) Environmental factors influence the production of enterobactin, salmochelin, aerobactin, and yersiniabactin in *Escherichia coli* strain Nissle 1917. Int J Med Microbiol 296: 513–520. 17008127 10.1016/j.ijmm.2006.06.003

[42] Lv HT, Hung CS, Henderson JP (2014) Metabolomic analysis of siderophore cheater mutants reveals metabolic costs of expression in uropathogenic *Escherichia coli* . J Prot Res 13: 1397–1404. 10.1021/pr4009749 PMC3993901 24476533

[43] Buckling A, Harrison F, Vos M, Brockhurst MA, Gardner A, et al (2007) Siderophore-mediated cooperation and virulence in *Pseudomonas aeruginosa* . FEMS Microbiol Ecol 62: 135–141. 17919300 10.1111/j.1574-6941.2007.00388.x

[44] De Vos D, De Chial M, Cochez C, Jansen S, Tummler B, et al (2001) Study of pyoverdine type and production by *Pseudomonas aeruginosa* isolated from cystic fibrosis patients: prevalence of type II pyoverdine isolates and accumulation of pyoverdine-negative mutations. Arch Microbiol 175: 384–388. 11409549 10.1007/s002030100278

[45] Kuemmerli R, Griffin AS, West SA, Buckling A, Harrison F (2009) Viscous medium promotes cooperation in the pathogenic bacterium *Pseudomonas aeruginosa* . Proc R Soc B 276: 3531–3538. 10.1098/rspb.2009.0861 19605393 PMC2817189

[46] Chen SL, Wu M, Henderson JP, Hooton TM, Hibbing ME, et al (2013) Genomic diversity and fitness of *E*. *coli* strains recovered from the intestinal and urinary tracts of women with recurrent urinary tract infection. Sci Transl Med 5: 184ra 160. 10.1126/scitranslmed.3005497 PMC3695744 23658245

[47] Russo TA, Stapleton A, Wenderoth S, Hooton TM, Stamm WE (1995) Chromosomal restrction-fragment-length-polymorphism analysis of *Escherichia coli* strains causing recurrent urinary-tract infections in young women. J Infect Dis 172: 440–445. 7622887 10.1093/infdis/172.2.440

