# Supplementary figures and images for: Variation in Siderophore Biosynthetic Gene Distribution and Production across Environmental and Faecal Populations of Escherichia coli
Source: PLoS One. 2015 Mar 10;10(3):e0117906. doi: 10.1371/journal.pone.0117906 (PMC4355413; doi:10.1371/journal.pone.0117906)

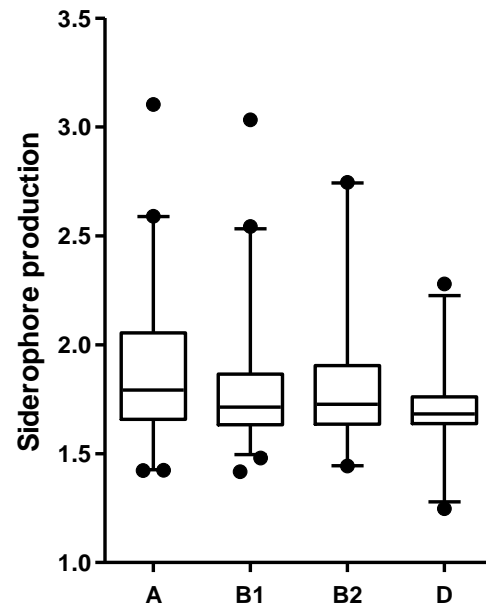

Supplement: S1 Fig — Box plots showing siderophore production by GMB and ECOR-F strains split according to the major E. coli phylogenetic groups. The central rectangle of the plot spans the interquartile range (IQR). The segment inside the rectangle shows the median, while the whiskers span the 5–95 percentile. Black circles represent outliers. Statistical significance was determined using the Student t-test. No significant differences were detected. (PDF) [file pone.0117906.s001.pdf]

**A**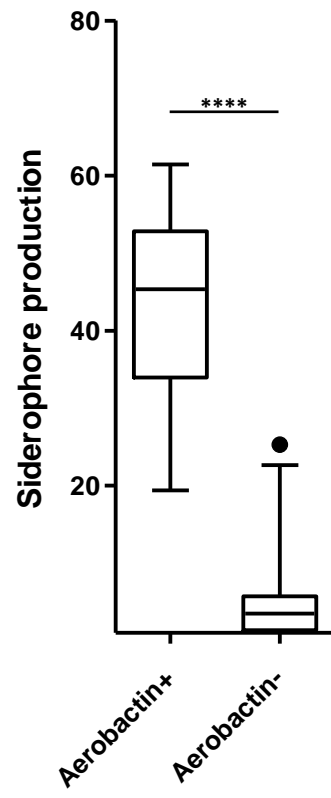**B**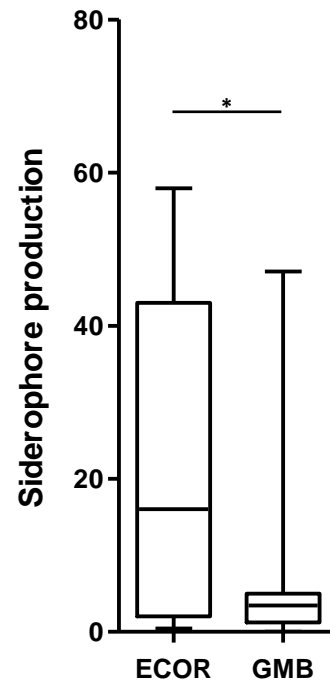

Supplement: S2 Fig — Box plots showing siderophore production in liquid CAS for a subset of strains (n = 33) to visualise differences in siderophore production of A) strains with or without the aerobactin biosynthesis locus, and B) GMB and ECOR-F. The central rectangle of the plot spans the interquartile range (IQR). The segment inside the rectangle shows the median, while the whiskers span the 5–95 percentile. Black circles represent outliers. Statistical significance was determined using the Student t-test; *P<0.05, ****P<0.0001. (PDF) [file pone.0117906.s002.pdf]
