# Supplementary material for: Variation in Siderophore Biosynthetic Gene Distribution and Production across Environmental and Faecal Populations of Escherichia coli
Source: PLoS One. 2015 Mar 10;10(3):e0117906. doi: 10.1371/journal.pone.0117906 (PMC4355413; doi:10.1371/journal.pone.0117906)
Supplement: S1 Table — The table displays the ratios between colony and halo diameter as measured on chrome azurol S agar plates for each GMB and ECOR-F strain. (PDF) [file pone.0117906.s003.pdf]

Supplementary Table S1

Strain	Siderophore production (d_{halo}/d_{colony})
ECOR05	3.104
ECOR70	3.033
ECOR37	2.840
ECOR51	2.746
ECOR39	2.741
ECOR63	2.721
ECOR41	2.656
ECOR24	2.590
ECOR02	2.560
ECOR38	2.545
GMB59	2.543
GMB88	2.530
GMB104	2.452
ECOR08	2.303
GMB03	2.282
GMB04	2.280
GMB91	2.274
ECOR06	2.171
ECOR55	2.163
ECOR07	2.160
ECOR20	2.155
ECOR30	2.149
ECOR17	2.076
GMB14	2.056
ECOR43	2.014
ECOR57	2.012
GMB15	2.007
GMB70	1.995
ECOR21	1.991
GMB01	1.991
ECOR59	1.987
ECOR28	1.982
ECOR09	1.977
ECOR67	1.970
GMB44	1.939
ECOR32	1.923
ECOR18	1.904
GMB05	1.901
GMB105	1.891
GMB84	1.890
GMB80	1.889
ECOR15	1.880
ECOR45	1.867
ECOR34	1.865
ECOR13	1.854
ECOR36	1.851

GMB23	1.845
GMB103	1.824
GMB76	1.823
GMB48	1.821
GMB43	1.815
ECOR12	1.800
GMB79	1.791
ECOR33	1.790
ECOR26	1.788
ECOR58	1.786
GMB28	1.786
GMB33	1.783
GMB89	1.783
GMB30	1.780
ECOR65	1.774
ECOR19	1.772
GMB101	1.767
GMB60	1.760
GMB97	1.760
GMB21	1.755
GMB81	1.750
ECOR27	1.748
GMB73	1.746
GMB77	1.743
GMB63	1.737
GMB94	1.735
GMB52	1.733
ECOR53	1.733
GMB47	1.726
GMB06	1.726
GMB74	1.726
GMB90	1.723
GMB87	1.722
GMB40	1.721
GMB45	1.719
GMB92	1.717
ECOR44	1.716
GMB99	1.716
GMB36	1.714
GMB95	1.709
ECOR31	1.708
GMB78	1.705
GMB10	1.705
ECOR04	1.701
GMB64	1.700
GMB107	1.697
GMB65	1.696
ECOR03	1.695
GMB98	1.690
GMB34	1.689

ECOR54	1.684
GMB72	1.683
GMB108	1.679
GMB38	1.678
GMB58	1.676
GMB49	1.673
GMB83	1.673
GMB35	1.670
ECOR35	1.666
ECOR66	1.663
ECOR25	1.661
GMB53	1.659
ECOR01	1.657
GMB67	1.655
GMB54	1.655
GMB29	1.651
GMB19	1.651
GMB61	1.649
ECOR69	1.648
GMB32	1.646
GMB71	1.644
GMB66	1.644
GMB68	1.637
GMB41	1.633
ECOR47	1.626
GMB93	1.608
GMB96	1.607
ECOR46	1.605
GMB16	1.602
GMB25	1.600
GMB13	1.589
GMB02	1.588
GMB17	1.580
ECOR23	1.579
ECOR16	1.579
GMB24	1.576
GMB100	1.573
GMB86	1.572
GMB39	1.568
ECOR10	1.566
GMB51	1.564
ECOR61	1.557
GMB26	1.544
GMB85	1.519
GMB07	1.508
GMB27	1.505
GMB18	1.500
ECOR68	1.480
GMB106	1.471
GMB102	1.460

ECOR42	1.458
GMB31	1.444
GMB20	1.424
ECOR22	1.422
GMB22	1.417
GMB50	1.405
ECOR49	1.248
ECOR29	n/a*
ECOR52	n/a*
GMB37	n/a*
GMB69	n/a*

*No growth could be observed on CAS agar plates
