# Supplementary material for: Variation in Siderophore Biosynthetic Gene Distribution and Production across Environmental and Faecal Populations of Escherichia coli
Source: PLoS One. 2015 Mar 10;10(3):e0117906. doi: 10.1371/journal.pone.0117906 (PMC4355413; doi:10.1371/journal.pone.0117906)
Supplement: S2 Table — The table shows presence/absence of the genes encoded in siderophore production loci in E. coli isolates from the ECOR strain collection. (PDF) [file pone.0117906.s004.pdf]

Supplementary Table S2

		Aerobactin					Enterobactin					Salmochelin					Yersiniabactin				
Strain	Phylogroup	*iucA*	*iucB*	*iucC*	*iucD*	*iutA*	*entA*	*entB*	*entC*	*entE*	*fepA*	*iroB*	*iroC*	*iroD*	*iroE*	*iroN*	*irp2*	*irp1*	*irp3*[#]	*irp4-5*[#]	*fyuA*
ECOR01	A	-	-	-	-	-	+	+	+	+	+	-	-	-	-	-	-	-	-	-	-
ECOR02	A	-[a]	-[a]	-[a]	-[a]	-[b]	+	+	+	+	+	-	-	-	-	-	+	+	+	+	+
ECOR03	A	-	-	-	-	-	+	+	+	+	+	-	-	-	-	-	-	-	-	-	-
ECOR04	A	-	-	-	-	-	+	+	+	+	+	-	-	-	-	-	-	-	-	-	-
ECOR05	A	+	+	+	+	+	+	+	+	+	+	-	+	+	+	-	-	-	-	-	-
ECOR06	A	-	-	-	-	-	+	+	+	+	+	-	-	-	-	-	-	-	-	-	-
ECOR07	A	+[a]	+	+	+	+[c]	+	+	+	+	+	+	+	+	+	+	+[d]	+[d]	+	+	+[d]
ECOR08	A	+	+	+	+	+	+	+	+	+	+	-	-	-	-	-	+	+	+	+	+
ECOR09	A	-	-	-[a]	-[a]	-[c]	+	+	+	+	+	-	-	-	-	-	+	+	+	+	+
ECOR10	A	-	-	-	-	-	+	+	+	+	+	-	-	-	-	-	+	+	+	+	+
ECOR11[e]	A	+	+	+	+	+[c]	+	+	+	+	+	-	-	-	-	-	+	+	+	+	+
ECOR12	A	-	-	-	-	-	+	+	+	+	+	-	-	-	-	-	-	-	-	-	-
ECOR13	A	-	-	-	-	-	+	+	+	+	+	-	-	-	-	-	-	-	-	-	-
ECOR14[e]	A	-	-	-	-	-	+	+	+	+	+	-	-	-	-	-	-	-	-	-	-
ECOR15	A	-	-	-	-	-	+	+	+	+	+	-	-	-	-	-	-	-	-	-	-
ECOR16	A	-	-	-	-	-	+	+	+	+	+	-	-	-	-	-	+	+	+	+	+
ECOR17	A	-	-	-	-	-	+	+	+	+	+	-	-	-	-	-	-	-	-	-	-
ECOR18	A	-	-	-	-	-	+	+	+	+	+	-	-	-	-	-	-	-	-	-	-
ECOR19	A	-	-	-	-	-	+	+	+	+	+	-	-	-	-	-	-	-	-	-	-
ECOR20	A	-	-	-	-	-	+	+	+	+	+	-	-	-	-	-	-	-	-	-	-
ECOR21	A	-	-	-	-	-	+	+	+	+	+	-	-	-	-	-	-	-	-	-	-
ECOR22	A	-	-	-	-	-	+	+	+	+	+	-	-	-	-	-	-	-	-	-	-
ECOR23	A	-	-	-	-	-	+	+	+	+	+	-	-	-	-	-	-	-	-	-	-
ECOR24	A	+	+	+	+	+	+	+	+	+	+	-	-	-	-	-	+	+	+	+	+
ECOR25	A	-	-	-	-	-	+	+	+	+	+	-	-	-	-	-	-	-	-	-	-
ECOR26	B1	-	-	-	-	-	+	+	+	+	+	-	-	-	-	-	-	-	-	-	-
ECOR27	B1	-	-	-	-	-	+	+	+	+	+	-	-	-	-	-	-	-	-	-	-
ECOR28	B1	-	-	-	-	-	+	+	+	+	+	-	-	-	-	-	-	-	-	-	-
ECOR29	B1	-	-	-	-	-	+	+	+	+	+	-	-	-	-	-	-	-	-	-	-
ECOR30	B1	-	-	-	-	-	+	+	+	+	+	+	+	+	+	+	-	-	-	-	-
ECOR31	E	-	-	-	-	-	+	+	+	+	+	-	-	-	-	-	+	+	+	+	+
ECOR32	B1	-	-	-	-	-	+	+	+	+	+	-[a]	-[a]	-[a]	-[a]	-[c]	-	-	-	-	-
ECOR33	B1	-	-	-	-	-	+	+	+	+	+	-	-	-	-	-	-	-	-	-	-
ECOR34	B1	-	-	-	-	-	+	+	+	+	+	-	-	-	-	-	-	-	-	-	-
ECOR35	F	+	+	+	+	+	+	+	+	+[a]	+	-	-	-	-	-	+	+	+	+	+
ECOR36	F	+	+	+	+	+	+	+	+	+[a]	+	-	-	-	-	-	+	+	+	+	+
ECOR37	E	+	+	+	+	+	+	+	+	+	+	-	-	-	-	-	-	-	-	-	-
ECOR38	F	+	+	+	+	+[c]	+	+	+	+	+	-	-	-	-	-	+	+	+	+	+
ECOR39	F	+	+	+	+	+[c]	+	+	+	+	+	-	-	-	-	-	+	+	+	+	+
ECOR40[e]	F	+	+	+	+	+[c]	+	+	+	+	+	-	-	-	-	-	+	+	+	+	+
ECOR41	F	+	+	+	+	+[c]	+	+	+	+	+	-	-	-	-	-	+	+	+	+	+
ECOR42	E	-	-	-	-	-	+	+	+	+	+	-	-	-	-	-	-	-	-	-	-
ECOR43	E	-	-	-	-	-	+	+	+	+	+	-	-	-	-	-	-	-	-	-	-
ECOR44	D	-	-	-	-	-	+	+	+	+	+	-	-	-	-	-	-	-	-	-	-
ECOR45	B1	-	-	-	-	-	+	+	+	+	+	-	-	-	-	-	-	-	-	-	-
ECOR46	D	-	-	-	-	-	+	+	+	+	+	-	-	-	-	-	+	+	+	+	+
ECOR47	D	-	-	-	-	-	+	+	+	+	+	-	-	-	-	-	-	-	-	-	-
ECOR48[e]	D	-	-	-	-	-	+	+	+	+	+	-	-	-	-	-	+	+	+	+	+
ECOR49	D	+	+	+	+	+[c]	+	+	+	+	+	-	-	-	-	-	+	+	+	+	+
ECOR50[e]	D	+	+	+	+	+[c]	+	+	+	+	+	-	+	+	+	-	+	+	+	+	+
ECOR51	B2	+	+	+	+	+[c]	+	+	+	+	+	+	+	+	+	+	+	+	+	+	+
ECOR52	B2	-	-	-	-	-	+	+	+	+	+	+	+	+	+	+	+	+	+	+	+
ECOR53	B2	-	-	-	-	-	+	+	+	+	+	+	+	+	+	+	+	+	+	+	+
ECOR54	B2	-	-	-	-	-	+	+	+	+	+	+	+	+	+	+	+	+	+	+	+
ECOR55	B2	+	+	+	+	+[c]	+	+	+	+	+	-	-	-	-	-	+	+	+	+	+
ECOR56[e]	B2	+	+	+	+	+	+	+	+	+	+	-	-	-	-	-	+	+	+	+	+
ECOR57	B2	+	+	+	+	+[c]	+	+	+	+	+	+	+	+	+	+	+	+	+	+	+
ECOR58	B1	-	-	-	-	-	+	+	+	+	+	+	+	+	+	+	-	-	-	-	-
ECOR59	B2	-	-	-	-	-	+	+	+	+	+	-	-[a]	-[a]	-[a]	-	+	+	+	+	+
ECOR60[e]	B2	-	-	-	-	-	+	+	+	+	+	+	+	+	+	+	+	+	+	+	+
ECOR61	B2	-	-	-	-	-	+	+	+	+	+	-	-	-	-	-	+	+	+	+	+
ECOR62[e]	B2	+[a]	+	+	+	+[c]	+	+	+	+	+	+	+	+	+	+	+	+	+	+	+
ECOR63	B2	-	-	-	-	-	+	+	+	+	+	+	+	+	+	+	+	+	+	+	+
ECOR64[e]	B2	-	-	-	-	-	+	+	+	+	+	+	+	+	+	+	+	+	+	+	+
ECOR65	B2	-	-	-	-	-	+	+	+	+	+	+	+	+	+	+	+	+	+	+	+
ECOR66	B2	-	-	-	-	-	+	+	+	+	+	+	+	+	+	+	+	+	+	+	+
ECOR67	B1	-	-	-	-	-	+	+	+	+	+	-[a]	-[a]	-[a]	-[a]	-[b]	-	-	-	-	-

ECOR68	B1	-	-	-	-	-	+	+	+	+	+	-	-	-	-	-	+	+	+	+	+
ECOR69	B1	-	-	-	-	-	+	+	+	+	+	-	-	-	-	-	-	-	-	-	-
ECOR70	B1	+[a]	+	+	+	+[c]	+	+	+	+	+	+	+	+	+	+	+	+	+	+	+
ECOR71[e]	B1	-	-	-	-	-	+	+	+	+	+	-	-	-	-	-	+	+	+	+	+
ECOR72[e]	B1	-	-	-	-	-	+	+	+	+	+	-	-	-	-	-	-[d]	-[d]	-	-	-[d]

+	Gene detected by multiplex-PCR in this study
-	Gene not detected by multiplex-PCR in this study

[a]Result different from array-based study (Jackson et al., 2011). Additional independent literature data not available.

[b]Result different from array-based and PCR-based studies (Johnson et al., 2001; Jackson et al., 2011).

[c]Result different from array-based study, but supported by PCR-based study (Johnson et al., 2001; Jackson et al., 2011).

[d]Result different from array-based and PCR-based studies (Schubert et al., 2009; Jackson et al., 2011).

[e]Strian isolated from the urine of women with urinary tract infections

[#]Independent literature data are not available for *irp3-5* .
