# Supplementary material for: Variation in Siderophore Biosynthetic Gene Distribution and Production across Environmental and Faecal Populations of Escherichia coli
Source: PLoS One. 2015 Mar 10;10(3):e0117906. doi: 10.1371/journal.pone.0117906 (PMC4355413; doi:10.1371/journal.pone.0117906)
Supplement: S3 Table — The table shows presence/absence of the genes encoded in siderophore production loci in E. coli isolates from the GMB strain collection. (PDF) [file pone.0117906.s005.pdf]

Supplementary Table S3

		Aerobactin					Enterobactin					Salmochelin					Yersiniabactin				
**Strain**	**Phylogroup**	***iucA***	***iucB***	***iucC***	***iucD***	***iutA***	***entA***	***entB***	***entC***	***entE***	***fepA***	***iroB***	***iroC***	***iroD***	***iroE***	***iroN***	***irp2***	***irp1***	***irp3***	***irp4-5***	***fyuA***
GMB01	A	-	-	-	-	-	+	+	+	+	+	-	-	-	-	-	-	-	-	-	-
GMB02	A	-	-	-	-	-	+	+	+	+	+	-	-	-	-	-	-	-	-	-	-
GMB03	B1	-	-	-	-	-	+	+	+	+	+	-	-	-	-	-	-	-	-	-	-
GMB04	D	-	-	-	-	-	+	+	+	+	+	-	-	-	-	-	-	-	-	-	-
GMB05	B1	-	-	-	-	-	+	+	+	+	+	-	-	-	-	-	-	-	-	-	-
GMB06	A	-	-	-	-	-	+	+	+	+	+	-	-	-	-	-	-	-	-	-	-
GMB07	B1	-	-	-	-	-	+	+	+	+	+	-	-	-	-	-	+	+	+	+	+
GMB10	B1	-	-	-	-	-	+	+	+	+	+	-	-	-	-	-	-	-	-	-	-
GMB100	B2	-	-	-	-	-	+	+	+	+	+	-	-	-	-	-	+	+	+	+	+
GMB101	B2	-	-	-	-	-	+	+	+	+	+	-	-	-	-	-	+	+	+	+	+
GMB102	B2	-	-	-	-	-	+	+	+	+	+	-	-	-	-	-	+	+	+	+	+
GMB103	B1	-	-	-	-	-	+	+	+	+	+	-	-	-	-	-	-	-	-	-	-
GMB104	A	+	+	+	+	+	+	+	+	+	+	+	+	+	+	+	+	+	+	+	+
GMB105	B1	-	-	-	-	-	+	+	+	+	+	+	+	+	+	+	-	-	-	-	-
GMB106	A	-	-	-	-	-	+	+	+	+	+	-	-	-	-	-	-	-	-	-	-
GMB107	B1	-	-	-	-	-	+	+	+	+	+	-	-	-	-	-	-	-	-	-	-
GMB108	A	-	-	-	-	-	+	+	+	+	+	-	-	-	-	-	-	-	-	-	-
GMB13	B1	-	-	-	-	-	+	+	+	+	+	-	-	-	-	-	-	-	-	-	-
GMB14	B1	-	-	-	-	-	+	+	+	+	+	-	-	-	-	-	-	-	-	-	-
GMB15	D	+	+	+	+	+	+	+	+	+	+	+	+	+	+	+	-	-	-	-	-
GMB16	B1	-	-	-	-	-	+	+	+	+	+	-	-	-	-	-	-	-	-	-	-
GMB17	B1	-	-	-	-	-	+	+	+	+	+	-	-	-	-	-	-	-	-	-	-
GMB18	B1	-	-	-	-	-	+	+	+	+	+	-	-	-	-	-	-	-	-	-	-
GMB19	D	-	-	-	-	-	+	+	+	+	+	-	-	-	-	-	-	-	-	-	-
GMB20	A	-	-	-	-	-	+	+	+	+	+	-	-	-	-	-	-	-	-	-	-
GMB21	D	-	-	-	-	-	+	+	+	+	+	+	+	+	+	+	-	-	-	-	-
GMB22	B1	-	-	-	-	-	+	+	+	+	+	-	-	-	-	-	-	-	-	-	-
GMB23	A	+	+	+	+	+	+	+	+	+	+	-	-	-	-	-	+	+	+	+	+
GMB24	B1	-	-	-	-	-	+	+	+	+	+	-	-	-	-	-	-	-	-	-	-
GMB25	B1	-	-	-	-	-	+	+	+	+	+	-	-	-	-	-	-	-	-	-	-
GMB26	B1	-	-	-	-	-	+	+	+	+	+	-	-	-	-	-	-	-	-	-	-
GMB27	A	-	-	-	-	-	+	+	+	+	+	-	-	-	-	-	-	-	-	-	-
GMB28	B1	-	-	-	-	-	+	+	+	+	+	-	-	-	-	-	-	-	-	-	-
GMB29	B1	-	-	-	-	-	+	+	+	+	+	-	-	-	-	-	-	-	-	-	-
GMB30	D	-	-	-	-	-	+	+	+	+	+	+	+	+	+	+	-	-	-	-	-
GMB31	B2	-	-	-	-	-	+	+	+	+	+	+	+	+	+	+	+	+	+	+	+
GMB32	A	-	-	-	-	-	+	+	+	+	+	-	-	-	-	-	-	-	-	-	-
GMB33	A	-	-	-	-	-	+	+	+	+	+	-	-	-	-	-	-	-	-	-	-
GMB34	A	-	-	-	-	-	+	+	+	+	+	-	-	-	-	-	-	-	-	-	-
GMB35	A	-	-	-	-	-	+	+	+	+	+	-	-	-	-	-	-	-	-	-	-
GMB36	B1	-	-	-	-	-	+	+	+	+	+	-	-	-	-	-	-	-	-	-	-
GMB37	A	-	-	-	-	-	+	+	+	+	+	-	-	-	-	-	-	-	-	-	-
GMB38	B1	-	-	-	-	-	+	+	+	+	+	-	-	-	-	-	-	-	-	-	-
GMB39	E	-	-	-	-	-	+	+	+	+	+	-	-	-	-	-	-	-	-	-	-
GMB40	B2	-	-	-	-	-	+	+	+	+	+	+	+	+	+	+	+	+	+	+	+
GMB41	B1	-	-	-	-	-	+	+	+	+	+	-	-	-	-	-	-	-	-	-	-
GMB43	B1	-	-	-	-	-	+	+	+	+	+	-	-	-	-	-	-	-	-	-	-
GMB44	D	-	-	-	-	-	+	+	+	+	+	-	-	-	-	-	-	-	-	-	-
GMB45	B2	-	-	-	-	-	+	+	+	+	+	-	-	-	-	-	+	+	+	+	+
GMB47	B2	-	-	-	-	-	+	+	+	+	+	-	-	-	-	-	+	+	+	+	+
GMB48	A	-	-	-	-	-	+	+	+	+	+	-	-	-	-	-	-	-	-	-	-
GMB49	B1	-	-	-	-	-	+	+	+	+	+	+	+	+	+	+	-	-	-	-	-
GMB50	D	-	-	-	-	-	+	+	+	+	+	-	-	-	-	-	-	-	-	-	-
GMB51	D	-	-	-	-	-	+	+	+	+	+	-	-	-	-	-	+	+	+	+	+
GMB52	D	-	-	-	-	-	+	+	+	+	+	-	-	-	-	-	-	-	-	-	-
GMB53	B1	-	-	-	-	-	+	+	+	+	+	-	-	-	-	-	+	+	+	+	+
GMB54	D	-	-	-	-	-	+	+	+	+	+	-	-	-	-	-	-	-	-	-	-
GMB58	B1	-	-	-	-	-	+	+	+	+	+	-	-	-	-	-	-	-	-	-	-
GMB59	B1	+	+	+	+	+	+	+	+	+	+	+	+	+	+	+	-	-	-	-	-
GMB60	D	-	-	-	-	-	+	+	+	+	+	-	-	-	-	-	-	-	-	-	-
GMB61	D	-	-	-	-	-	+	+	+	+	+	-	-	-	-	-	-	-	-	-	-
GMB63	D	-	-	-	-	-	+	+	+	+	+	-	-	-	-	-	-	-	-	-	-
GMB64	B1	-	-	-	-	-	+	+	+	+	+	-	-	-	-	-	-	-	-	-	-
GMB65	B1	-	-	-	-	-	+	+	+	+	+	-	-	-	-	-	-	-	-	-	-
GMB66	D	-	-	-	-	-	+	+	+	+	+	-	-	-	-	-	-	-	-	-	-
GMB67	B1	-	-	-	-	-	+	+	+	+	+	-	-	-	-	-	-	-	-	-	-
GMB68	D	-	-	-	-	-	+	+	+	+	+	-	-	-	-	-	-	-	-	-	-
GMB69	B1	-	-	-	-	-	+	+	+	+	+	-	-	-	-	-	-	-	-	-	-
GMB70	D	-	-	-	-	-	+	+	+	+	+	-	-	-	-	-	-	-	-	-	-
GMB71	D	-	-	-	-	-	+	+	+	+	+	-	-	-	-	-	-	-	-	-	-
GMB72	D	-	-	-	-	-	+	+	+	+	+	-	-	-	-	-	-	-	-	-	-
GMB73	B2	-	-	-	-	-	+	+	+	+	+	+	+	+	+	+	+	+	+	+	+
GMB74	D	-	-	-	-	-	+	+	+	+	+	-	-	-	-	-	-	-	-	-	-
GMB76	B2	-	-	-	-	-	+	+	+	+	+	+	+	+	+	+	+	+	+	+	+

GMB77	E	-	-	-	-	-	+	+	+	+	+	-	-	-	-	-	-	-	-	-	-
GMB78	E	-	-	-	-	-	+	+	+	+	+	-	-	-	-	-	-	-	-	-	-
GMB79	B1	-	-	-	-	-	+	+	+	+	+	-	-	-	-	-	-	-	-	-	-
GMB80	B1	-	-	-	-	-	+	+	+	+	+	-	-	-	-	-	-	-	-	-	-
GMB81	B1	-	-	-	-	-	+	+	+	+	+	-	-	-	-	-	-	-	-	-	-
GMB83	B1	-	-	-	-	-	+	+	+	+	+	-	-	-	-	-	-	-	-	-	-
GMB84	A	-	-	-	-	-	+	+	+	+	+	-	-	-	-	-	-	-	-	-	-
GMB85	B1	-	-	-	-	-	+	+	+	+	+	-	-	-	-	-	-	-	-	-	-
GMB86	B1	-	-	-	-	-	+	+	+	+	+	-	-	-	-	-	-	-	-	-	-
GMB87	E	-	-	-	-	-	+	+	+	+	+	-	-	-	-	-	+	+	+	+	+
GMB88	B1	+	+	+	+	+	+	+	+	+	+	+	+	+	+	+	-	-	-	-	-
GMB89	B1	-	-	-	-	-	+	+	+	+	+	-	-	-	-	-	-	-	-	-	-
GMB90	A	-	-	-	-	-	+	+	+	+	+	-	-	-	-	-	-	-	-	-	-
GMB91	A	-	-	-	-	-	+	+	+	+	+	-	-	-	-	-	-	-	-	-	-
GMB92	B1	-	-	-	-	-	+	+	+	+	+	-	-	-	-	-	-	-	-	-	-
GMB93	B2	-	-	-	-	-	+	+	+	+	+	+	+	+	+	+	+	+	+	+	+
GMB94	B1	-	-	-	-	-	+	+	+	+	+	-	-	-	-	-	-	-	-	-	-
GMB95	B1	-	-	-	-	-	+	+	+	+	+	-	-	-	-	-	-	-	-	-	-
GMB96	B1	-	-	-	-	-	+	+	+	+	+	-	-	-	-	-	-	-	-	-	-
GMB97	E	-	-	-	-	-	+	+	+	+	+	-	-	-	-	-	+	+	+	+	+
GMB98	B2	-	-	-	-	-	+	+	+	+	+	+	+	+	+	+	+	+	+	+	+
GMB99	B1	-	-	-	-	-	+	+	+	+	+	-	-	-	-	-	-	-	-	-	-

+	Gene detected by multiplex-PCR in this study
-	Gene not detected by multiplex-PCR in this study
